# Supplementary material for: The causal relationship between the human gut microbiota and pyogenic arthritis: a Mendelian randomization study
Source: Front Cell Infect Microbiol. 2024 Nov 26;14:1452480. doi: 10.3389/fcimb.2024.1452480 (PMC11629706; doi:10.3389/fcimb.2024.1452480)
Supplement: Supplementary file 1 [file DataSheet1.zip › mendelian test/ebi-a-GCST90027546.csv_forest.pdf]

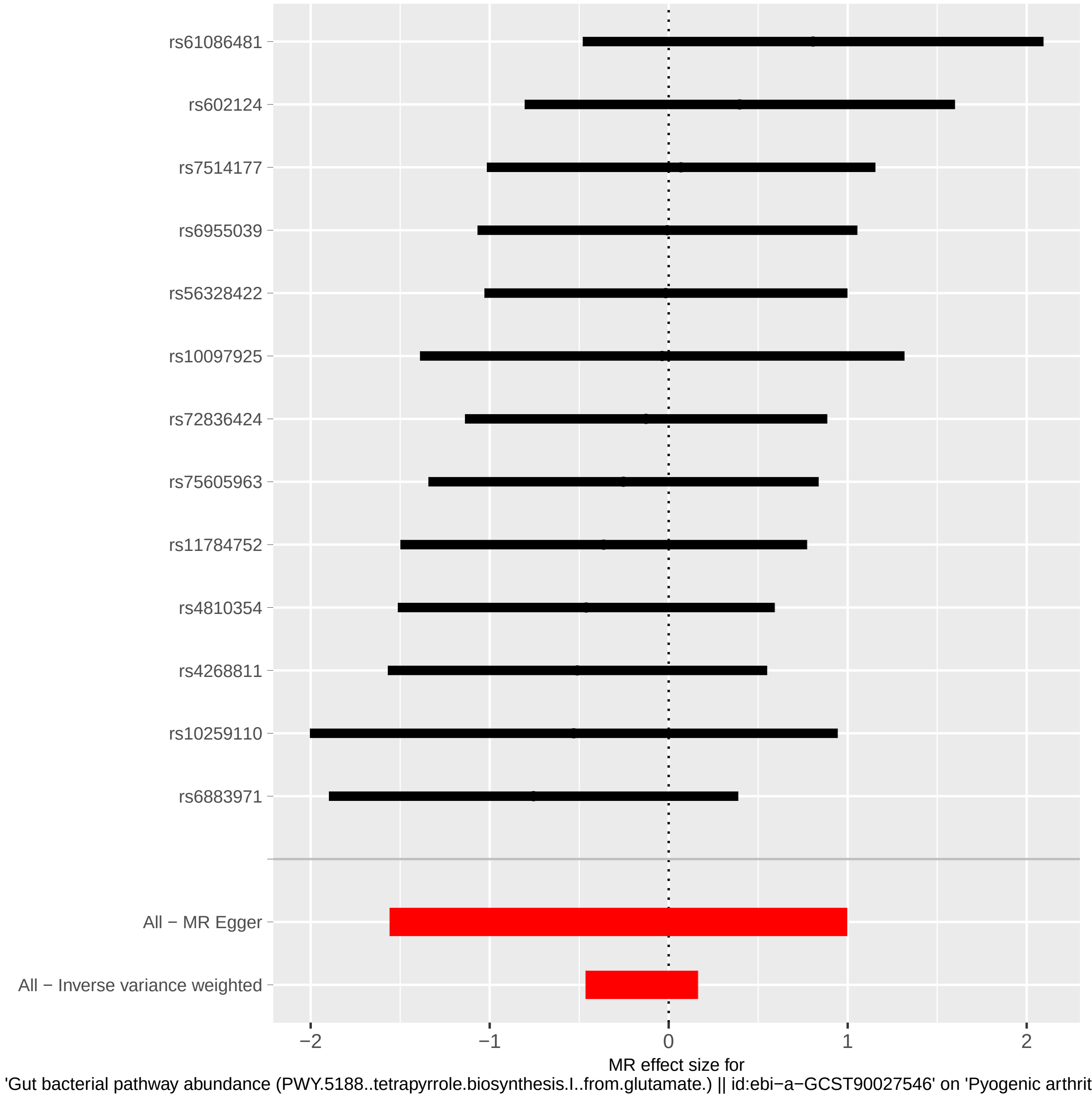
rs61086481
rs602124
rs7514177
rs6955039
rs56328422
rs10097925
rs72836424
rs75605963
rs11784752
rs4810354
rs4268811
rs10259110
rs6883971
All − MR Egger
All − Inverse variance weighted
−2
−1
0
1
2
MR effect size for
'Gut bacterial pathway abundance (PWY.5188..tetrapyrrole.biosynthesis.I..from.glutamate.) || id:ebi−a−GCST90027546' on 'Pyogenic arthrit
